# Development and validation of a multivariable prediction model for non-invasive discrimination between diabetic and non-diabetic kidney disease in type 2 diabetes: a clinical nomogram

**DOI:** 10.3389/fendo.2026.1787412

**Published:** 2026-03-05

**Authors:** Lin Li, Fuzhe Ma, Chaonan Bao, Tao Sun, Shaojie Fu, Zhonggao Xu

**Affiliations:** 1 Department of Nephrology, The First Hospital of Jilin University, Changchun, China; 2 Department of Gastroenterology, The First Hospital of Jilin University, Changchun, China

**Keywords:** diabetes complications, diabetes mellitus, type 2, diagnosis, kidney diseases, nomogram

## Abstract

**Objective:**

This study aimed to develop a non-invasive diagnostic model to differentiate diabetic kidney disease (DKD) from non-diabetic kidney disease (NDKD) in type 2 diabetes mellitus (T2DM) patients with renal insufficiency.

**Methods:**

We conducted a retrospective, biopsy-based study of diabetic patients with kidney dysfunction between July 2018 and August 2023. Patients were randomly split into training and validation cohorts (7:3). A multivariable logistic regression model based on routinely available, non-invasive clinical variables was developed and internally validated. Discrimination and calibration were evaluated in both cohorts.

**Results:**

A total of 507 patients were enrolled: 171 with DKD, 260 with NDKD, and 76 with concurrent DKD and NDKD. A five-variable model incorporating diabetes duration, diabetic retinopathy, systolic blood pressure, fasting plasma glucose, and hemoglobin levels demonstrated good discrimination and acceptable calibration in both datasets. Decision curve analysis suggested the model’s potential clinical utility. The model was presented as a nomogram.

**Conclusions:**

This nomogram may support non-invasive differential diagnosis between DKD and NDKD in T2DM patients with kidney injury, thereby informing clinical decision-making.

## 1 Introduction

With the rapid increase in the prevalence of diabetes, diabetic kidney disease (DKD), a common microvascular complication of diabetes, is emerging as the leading cause of end-stage kidney disease (1, 2). In 2019, the global burden of DKD was estimated at 13.09 million disability-adjusted life years (DALYs), with 75.38% of these DALYs attributed to DKD associated with type 2 diabetes mellitus (T2DM) (3). Furthermore, the presence of microalbuminuria in individuals with diabetes is associated with a 97% higher risk of cardiovascular disease and a 15% increase in all-cause mortality (4). Therefore, early identification is crucial for improving the prognosis of patients with DKD.

Currently, the diagnosis of DKD primarily follows the 2007 Kidney Disease Outcomes Quality Initiative (KDOQI) clinical practice guidelines (5). Nevertheless, studies have shown that predominantly relying on traditional clinical parameters from these guidelines, such as duration of diabetes, albumin excretion rate, and estimated glomerular filtration rate (eGFR), to distinguish DKD from non-diabetic kidney disease (NDKD) results in a specificity of merely 40.6% (6). The prevalence of NDKD among patients with T2DM and renal impairment is remarkably high, ranging from 33.0% to 72.5% (7). Intriguingly, recent research has identified heterogeneous clinical phenotypes of DKD. Diabetic patients with no clinically significant albuminuria (urinary albumin excretion [UAE] <30 mg/24h) but with impaired renal function (eGFR<60 mL/min/1.73m^2^) who progress to DKD are diagnosed with normoalbuminuric diabetic kidney disease (NADKD) (8). In some instances, patients with DKD may experience a decline in renal function before overt albuminuria develops (9). Additionally, microalbuminuria may even regress in a subset of diabetic patients (10). This phenotypic heterogeneity complicates the etiologic attribution of kidney disease in T2DM when relying solely on conventional clinical indicators.

We conducted a retrospective study of patients with T2DM and biopsy-confirmed kidney disease at our center. Using these data, we developed and internally validated a diagnostic model for DKD.

## 2 Materials and methods

### 2.1 Study population

**Figure 1:**
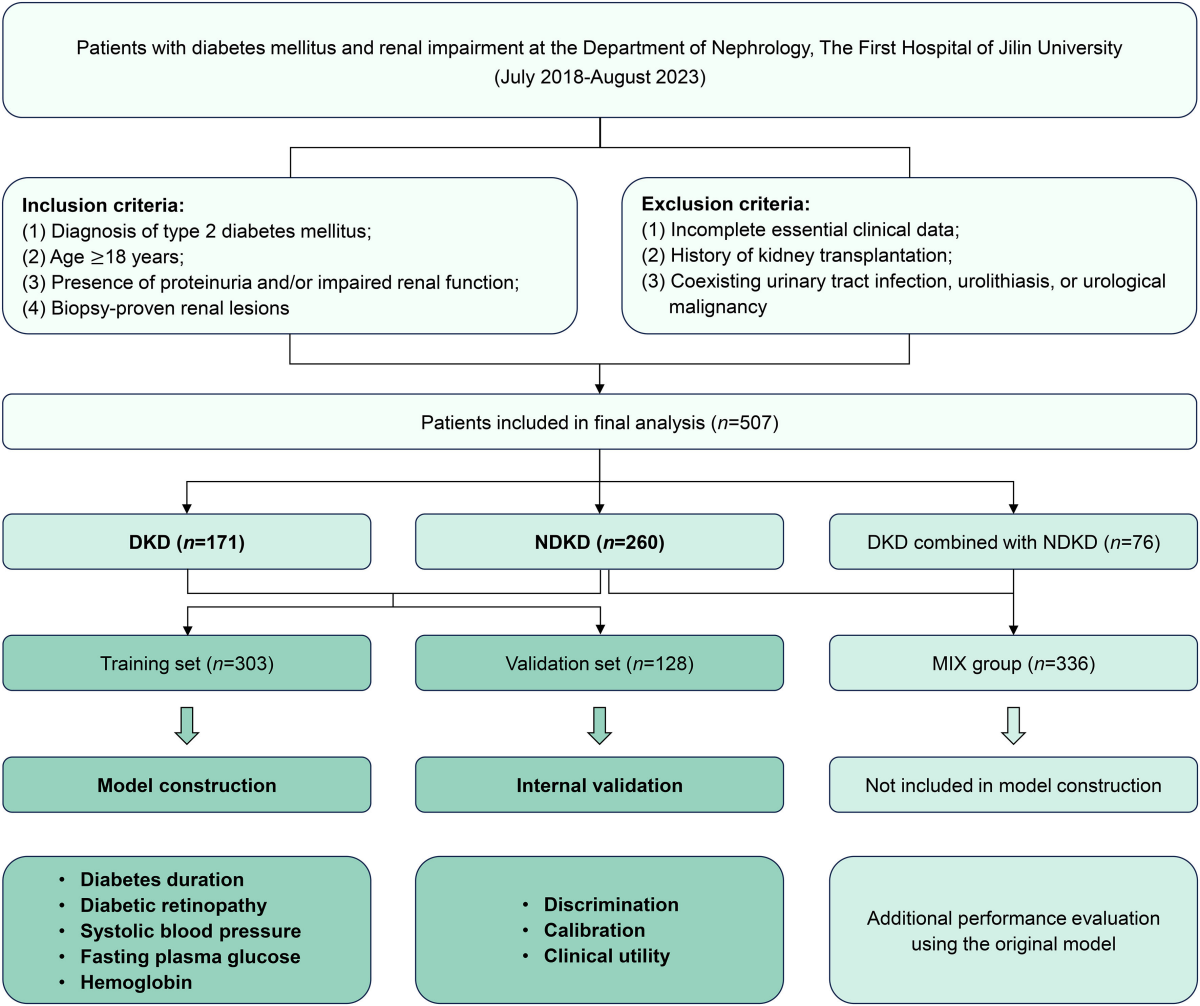
Study workflow. DKD, diabetic kidney disease; MIX group: NDKD and DKD combined with NDKD groups; NDKD, non-diabetic kidney disease.

This retrospective cohort study was conducted at the Department of Nephrology, The First Hospital of Jilin University. Patients with diabetes mellitus and renal impairment who underwent renal biopsy between July 2018 and August 2023 were enrolled. The study protocol received approval from the Ethics Committee of The First Hospital of Jilin University (Approval No. 2023-543). Written informed consent was obtained from all participants before they underwent renal biopsy procedures. The inclusion criteria were as follows: (1) diagnosis of T2DM according to the American Diabetes Association standards (11); (2) age of 18 years or older; (3) presence of proteinuria (defined as urinary protein excretion [UPE]>0.15 g/24h) and/or impaired renal function (defined as eGFR<90 mL/min/1.73m²); (4) biopsy-proven renal lesions. The exclusion criteria included: (1) incomplete essential clinical data; (2) a history of kidney transplantation; (3) coexisting urinary tract infection, urolithiasis, or urological malignancy. The flowchart of patient screening is summarized in **Supplementary Figure 1**. This study was conducted and reported in accordance with the Transparent Reporting of a Multivariable Prediction Model for Individual Prognosis or Diagnosis (TRIPOD) guidelines (12). The study workflow is summarized in **Figure 1**.

### 2.2 Data collection

The 32 predictive variables included in the study were selected based on clinical expertise and existing literature on the diagnosis of DKD (13–18). These variables encompass demographic characteristics, past medical history, relevant clinical examinations, and laboratory parameters. We collected the aforementioned indicators, such as sex, age, duration of diabetes and hypertension, smoking status, SBP, DBP, body mass index (BMI) (defined as weight in kilograms divided by the square of height in meters [kg/m²]), and DR (confirmed by standardized fundus photography conducted and interpreted by board-certified ophthalmologists with specialized training in retinal diseases). Hypertension was defined as systolic blood pressure (SBP)≥140 mmHg and/or diastolic blood pressure (DBP)≥90 mmHg, based on three separate office measurements taken on different days without antihypertensive medication use (1 mmHg=0.133 kPa) (19). Renal function was evaluated by measuring serum creatinine, cystatin C, retinol-binding protein (RBP), blood urea nitrogen (BUN), and eGFR, which was calculated using the Chronic Kidney Disease Epidemiology Collaboration (CKD-EPI) equation (20). Urinary parameters included 24-hour UPE, 24-hour UAE, urinary immunoglobulin G (IgG), urinary β2-microglobulin (β2-MG), and microscopic hematuria (defined as the presence of ≥3 red blood cells per high-power field). Metabolic profiles included fasting plasma glucose (FPG), glycated hemoglobin (HbA1c), triglycerides, total cholesterol, low-density lipoprotein cholesterol (LDL-C), and high-density lipoprotein cholesterol (HDL-C). Inflammatory markers included high-sensitivity C-reactive protein (Hs-CRP), erythrocyte sedimentation rate (ESR), and fibrinogen. Hematological indices consisted of hemoglobin (Hb), serum albumin, globulin, and uric acid. The durations of diabetes and hypertension were assessed from the initial diagnosis to hospital admission for renal biopsy. All of these parameters were evaluated during hospitalization for renal biopsy.

### 2.3 Renal outcomes

Renal biopsy procedures were carried out by experienced nephrologists under real-time ultrasound guidance during the patient’s hospitalization. The indications for renal biopsy in suspected NDKD at our center follow the criteria outlined in the 2007 KDOQI guidelines (5). Tissue samples were processed using standard procedures for both light microscopy and immunofluorescence analysis. Electron microscopy examinations were performed when necessary. For DKD diagnosis, biopsy samples must contain at least 10 complete glomeruli, excluding those located at the biopsy edges. The pathological diagnosis of DKD followed the 2010 Renal Pathology Society classification system (21). DKD is classified into four stages: Class I: Glomerular basement membrane thickening; Class II: Mesangial expansion; Class III: Nodular sclerosis (Kimmelstiel-Wilson lesions); and Class IV: Advanced diabetic glomerulosclerosis. The renal pathological classification adhered to the 1995 World Health Organization (WHO) classification of glomerular diseases, which includes primary glomerular diseases, secondary glomerular diseases, vascular glomerular diseases, and tubulointerstitial nephritis. The pathological type identified on renal biopsy, specifically DKD, was used as the outcome variable. All pathological diagnoses were independently reviewed and confirmed by at least two renal pathologists in collaboration with two nephrologists, and a consensus was reached on the final diagnosis.

### 2.4 Model construction and validation

For model development and validation, patients from the DKD and the NDKD groups were randomly assigned to the training and validation cohorts in a 7:3 ratio, as previously described (22). The model was developed using univariate and multivariable logistic regression analyses in the training cohort. 32 candidate predictors were initially screened using univariate logistic regression. Variables that reached statistical significance (*P<*0.05) in the univariate analysis (15 candidates in total) were subsequently entered into multivariable logistic regression to identify independent predictors of DKD. Variable selection was performed using forward stepwise selection based on the likelihood ratio test (Forward: LR), with a prespecified entry criterion of *P<*0.05 and a removal criterion of >0.10. This rigorous process identified five independent predictors. To ensure the stability and integrity of the model, multicollinearity was assessed by calculating the variance inflation factor (VIF) for each predictor. A VIF<5 was considered to indicate the absence of substantial multicollinearity among the retained predictors. For multivariable analyses, including logistic regression and factor analysis, we applied the commonly used rule of thumb stating that the sample size should be at least 10 times the number of variables. Given 32 candidate predictors, a sample size of 320 can be regarded as acceptable (23).

Internal validation was performed using the validation cohort. This approach ensures that the validation set remains relatively independent of the training process, which is crucial for evaluating the model’s generalizability to unseen data (12). Bootstrap internal validation with 1000 resamples was performed in the training cohort to assess the potential overfitting. Additionally, we conducted validation in the NDKD and the DKD combined with NDKD groups (MIX group). Model performance was assessed using the *C*-statistic, the Hosmer-Lemeshow goodness-of-fit test, and calibration curves in all datasets. The cutoff value was determined in the training cohort using the maximum Youden index and was prespecified as 0.370. This threshold was then applied unchanged to the validation cohort to classify patients (≥0.370 as high-risk, <0.370 as low-risk). Decision curve analysis was employed to verify the clinical applicability of the model, which was ultimately presented as a nomogram.

### 2.5 Statistical analysis

Data analyses were conducted with SPSS 27.0 software (SPSS Inc., Chicago, IL, USA) and R software version 4.5.1 (https://www.R-project.org). Non-normally distributed continuous data were reported as median (interquartile range [IQR]). Categorical variables were summarized using frequency and percentage. Appropriate statistical methods were employed for group comparisons: the Mann-Whitney *U* test and the Kruskal-Wallis *H* test for non-normally distributed variables, and the chi-square test for categorical variables, with Bonferroni correction applied in *post-hoc* multiple comparisons. Odds ratio (OR) was calculated and presented with 95% confidence intervals (CI). Missing data were evaluated using Little’s test for Missing Completely at Random (MCAR) and handled using multiple imputation by chained equations (MICE) via the “mice” package. The detailed distribution and processing of missing data patterns are presented in **Supplementary Figure 2**, **3**. Sensitivity analyses were conducted across all five imputed datasets (**Supplementary Table 1**, **2**). Receiver operating characteristic (ROC) and calibration (method=“boot”, B = 1000) curves were plotted using the “pROC” and “rms” packages. Diagnostic performance metrics, including sensitivity, specificity, positive predictive value (PPV), negative predictive value (NPV), accuracy, positive likelihood ratio (LR+), and negative likelihood ratio (LR–), were calculated at the optimal cutoff value using the “pROC” package. The Hosmer-Lemeshow test and decision curve analysis were assessed using the “ResourceSelection” and “rmda” packages. The final model was visualized as a nomogram using the “rms” package. Statistical significance was defined as a two-tailed *P* value<0.05.

## 3 Results

### 3.1 Study population and baseline characteristics

This study enrolled 507 participants diagnosed with T2DM and kidney dysfunction. Among them, 299 were male (59%), and the median age was 55 years (IQR: 45–61). The median duration of diabetes was 48 months (IQR: 6–120). DR was detected in 168 patients (33.1%). The median HbA1c was 7.0% (IQR: 6.2–8.0). The median eGFR was 65.00 mL/min/1.73m² (IQR: 39.67–91.06), and the median 24-hour UAE was 3316.60 mg/24h (IQR: 1400.27–5869.84). According to the renal biopsy results, patients were divided into three categories: DKD alone (*n=*171, 33.7%), NDKD alone (*n=*260, 51.3%), and DKD combined with NDKD (*n=*76, 15.0%).

Patients in both the DKD group and the DKD combined with NDKD group exhibited significantly longer diabetes duration, a higher prevalence of DR, and higher SBP levels compared to those in the NDKD group (*P<*0.05). The DKD group had the highest FPG levels, but the lowest Hb and eGFR levels (*P<*0.05). In contrast, the NDKD group exhibited the highest Hb levels and the lowest 24-hour UAE levels (*P<*0.05). **Table 1** displays a comparative evaluation of the clinical features and laboratory findings across the three groups.

**Table 1:** Clinical characteristics of patients with T2DM and renal impairment.

Characteristics	NDKD (*n=*260)	DKD (*n=*171)	DKD+NDKD (*n=*76)	*P* value
Sex (male, %)	159 (61.2)	100 (58.5)	40 (52.6)	0.408
Age (years)	55 (45, 62)	53 (42, 59)	58 (48, 63)^b^	0.030
BMI (kg/m^2^)	26.0 (23.9, 28.8)	25.4 (23.1, 27.9)	26.2 (24.2, 28.1)	0.173
Diabetes duration (months)	12 (1, 60)	120 (48, 192)^a^	96 (24, 135)^a^	<0.001
Diabetic retinopathy (%)	36 (13.8)	99 (57.9)^a^	33 (43.4)^a^	<0.001
Hypertension (months)	12 (0, 72)	12 (2, 60)	12 (1, 84)	0.261
SBP (mmHg)	139 (130, 151)	149 (135, 161)^a^	148 (137, 160)^a^	<0.001
DBP (mmHg)	82 (75, 90)	81 (75, 90)	83 (75, 90)	0.910
Smoker (%)	58 (22.3)	32 (18.7)	25 (32.9)^b^	0.048
FPG (mmol/L)	6.16 (5.20, 7.36)	6.90 (5.38, 9.20)^a^	5.88 (4.90, 7.69)^b^	0.001
HbA1c (%)	6.8 (6.2, 7.7)	7.3 (6.4, 9.0)^a^	6.9 (6.4, 7.7)	0.008
Hb (g/L)	137 (122, 150)	115 (97, 132)^a^	125 (109, 142)^ab^	<0.001
Hs-CRP (mg/L)	3.02 (1.65, 6.07)	2.41 (1.46, 4.31)	2.70 (1.57, 4.97)	0.170
ESR (mm/h)	38 (24, 56)	46 (27, 71)^a^	53 (28, 78)^a^	0.002
Fibrinogen (g/L)	4.19 (3.34, 5.45)	4.18 (3.62, 5.57)	5.04 (3.88, 6.38)^a^	0.004
Serum albumin (g/L)	30.0 (22.7, 36.8)	30.1 (24.9, 34.3)	24.3 (20.4, 29.0)^ab^	<0.001
Serum globulin (g/L)	26.1 (23.0, 29.4)	26.1 (23.7, 29.7)	24.7 (21.6, 27.2)^ab^	0.006
BUN (mmol/L)	6.30 (4.91, 8.45)	8.53 (6.44, 11.83)^a^	7.30 (5.99, 9.46)^a^	<0.001
Serum creatinine (μmol/L)	88.4 (67.0, 127.5)	130.2 (93.9, 189.9)^a^	99.9 (74.1, 136.6)^b^	<0.001
Serum RBP (mg/L)	57.9 (46.6, 75.9)	66.0 (52.5, 84.2)^a^	58.9 (44.4, 70.7)^b^	0.002
Serum cystatin C (mg/L)	1.13 (0.93, 1.68)	1.87 (1.25, 2.46)^a^	1.42 (1.11, 1.97)^a^	<0.001
Serum uric acid (μmol/L)	388 (321, 462)	411 (339, 471)	396 (339, 470)	0.259
eGFR (mL/min/1.73m^2^)	79.00 (52.00, 99.25)	50.00 (31.00, 69.00)^a^	63.50 (46.16, 85.56)^b^	<0.001
Triglycerides (mmol/L)	2.26 (1.57, 3.51)	2.02 (1.33, 3.12)	2.40 (1.68, 3.28)	0.034
Total cholesterol (mmol/L)	5.84 (4.64, 7.88)	5.67 (4.80, 7.23)	7.12 (5.67, 8.57)^ab^	<0.001
LDL-C (mmol/L)	3.54 (2.90, 4.86)	3.59 (2.80, 4.44)	4.65 (3.48, 5.60)^ab^	<0.001
HDL-C (mmol/L)	1.19 (0.97, 1.51)	1.21 (1.03, 1.48)	1.29 (1.14, 1.67)^a^	0.033
Microscopic hematuria (%)	152 (58.5)	89 (52.0)	51 (67.1)	0.080
UPE (g/24h)	2.940 (1.280, 7.480)	4.610 (2.550, 7.510)^a^	6.940 (4.470, 10.300)^ab^	<0.001
UAE (mg/24h)	2324.72(842.20,5584.50)	3657.50(1939.63,5415.49)^a^	4855.41(3132.20,7603.15)^ab^	<0.001
Urinary IgG (mg/24h)	143.60 (59.84, 451.58)	436.50 (180.47, 739.15)^a^	445.77 (167.66, 860.41)^a^	<0.001
Urinary β2-MG (mg/24h)	0.42 (0.14, 4.07)	4.19 (0.86, 19.13)^a^	2.27 (0.39, 11.67)^a^	<0.001

Data are presented as median (IQR) for skewed continuous variables and as proportions for categorical variables. The Kruskal-Wallis *H* test was used to compare non-normally distributed continuous variables across groups, and the chi-square test was used for categorical variables. Bonferroni correction applied for *post-hoc* multiple comparisons. β2-MG, β2-microglobulin; BMI, body mass index; BUN, blood urea nitrogen; DBP, diastolic blood pressure; DKD, diabetic kidney disease; DKD+NDKD, DKD combined with NDKD; eGFR, estimated glomerular filtration rate; ESR, erythrocyte sedimentation rate; FPG, fasting plasma glucose; Hb, hemoglobin; HbA1c, glycosylated hemoglobin; HDL-C, high-density lipoprotein cholesterol; Hs-CRP, high-sensitivity C-reactive protein; IgG, immunoglobulin G; IQR, interquartile range; LDL-C, low-density lipoprotein cholesterol; NDKD, non-diabetic kidney disease; RBP, retinol-binding protein; SBP, systolic blood pressure; T2DM, type 2 diabetes mellitus; UAE, urinary albumin excretion; UPE, urinary protein excretion. a: *P<*0.05 versus NDKD; b: *P<*0.05 versus DKD.

### 3.2 Renal pathological distributions

Membranous nephropathy emerged as the predominant pathological type in both the NDKD and the DKD combined with NDKD groups, accounting for 39.6% and 47.4%, respectively. IgA nephropathy ranked second, accounting for 20.8% and 17.1%, respectively. In the NDKD group, the most common secondary glomerular disease was Henoch-Schönlein purpura nephritis (3.8%), followed by obesity-associated glomerulomegaly (2.3%). In the DKD combined with NDKD group, hepatitis B virus-associated glomerulonephritis and immune complex-mediated glomerulonephritis (both at 5.3%) were the most frequently observed secondary glomerular diseases, followed by Henoch-Schönlein purpura nephritis and podocytopathy (both at 2.6%). **Table 2** presents the renal pathological findings in the NDKD and DKD combined with NDKD groups.

**Table 2:** Distribution of renal pathological types in the NDKD group and in the DKD combined with NDKD group.

Pathological types	NDKD (%)	DKD+NDKD (%)	Total (%)
Primary glomerular disease			
Membranous nephropathy	103 (39.6)	36 (47.4)	139 (41.4)
IgA nephropathy	54 (20.8)	13 (17.1)	67 (19.9)
Minimal change disease	14 (5.4)	2 (2.6)	16 (4.8)
Glomerular minor lesion	10 (3.8)	0 (0)	10 (3.0)
FSGS	5 (1.9)	2 (2.6)	7 (2.1)
MsPGN	3 (1.2)	1 (1.3)	4 (1.2)
MPGN	3 (1.2)	1 (1.3)	4 (1.2)
Endocapillary proliferative glomerulonephritis	2 (0.8)	0 (0)	2 (0.6)
Diffuse proliferative-sclerosing glomerulonephritis	2 (0.8)	0 (0)	2 (0.6)
Focal proliferative glomerulonephritis	1 (0.4)	0 (0)	1 (0.3)
Total	197 (75.8)	55 (72.4)	252 (75.0)
Secondary glomerular disease			
Henoch-Schönlein purpura nephritis	10 (3.8)	2 (2.6)	12 (3.6)
Obesity-associated glomerulomegaly	6 (2.3)	0 (0)	6 (1.8)
ANCA-associated glomerulonephritis	5 (1.9)	0 (0)	5 (1.5)
Cryoglobulinemia nephropathy	4 (1.5)	1 (1.3)	5 (1.5)
Lupus nephritis	3 (1.2)	1 (1.3)	4 (1.2)
Podocytopathy	3 (1.2)	2 (2.6)	5 (1.5)
Amyloid nephropathy	3 (1.2)	0 (0)	3 (0.9)
Hepatitis B virus-associated glomerulonephritis	1 (0.4)	4 (5.3)	5 (1.5)
C1q nephropathy	1 (0.4)	0 (0)	1 (0.3)
Immune complex-mediated glomerulonephritis	0 (0)	4 (5.3)	4 (1.2)
Monoclonal immunoglobulin deposition disease	0 (0)	1 (1.3)	1 (0.3)
Total	36 (13.8)	15 (19.7)	51 (15.2)
Renal vascular disease			
Ischemic renal injury	9 (3.5)	5 (6.6)	14 (4.2)
Thrombotic microangiopathy	2 (0.8)	0 (0)	2 (0.6)
Total	11 (4.2)	5 (6.6)	16 (4.8)
Tubulointerstitial nephritis	16 (6.2)	1 (1.3)	17 (5.1)
Total	260 (100.0)	76 (100.0)	336 (100.0)

ANCA, anti-neutrophil cytoplasmic antibody; DKD, diabetic kidney disease; DKD+NDKD, DKD combined with NDKD; NDKD, non-diabetic kidney disease; FSGS, focal segmental glomerulosclerosis; MPGN, membranoproliferative glomerulonephritis; MsPGN, mesangial proliferative glomerulonephritis.

### 3.3 Model development based on logistic regression analysis

Among 431 patients from the DKD and NDKD groups, random sampling was conducted in a 7 to 3 ratio, resulting in a training cohort (*n=*303) and a validation cohort (*n=*128). The proportion of DKD was 39.3% in the training cohort and 40.6% in the validation cohort. No statistically significant differences were observed between the two groups regarding demographic features, clinical examinations, or laboratory results (*P>*0.05), ensuring a balanced distribution for model development. **Supplementary Table 3** provides an overview of the baseline characteristics for both the training and validation cohorts.

Univariate logistic regression analysis conducted in the training cohort identified 15 significant variables associated with DKD (*P<*0.05). These variables included BMI, diabetes duration, DR, SBP, FPG, HbA1c, Hb, BUN, serum RBP, serum cystatin C, eGFR, total cholesterol, LDL-C, urinary IgG, and urinary β2-MG. Compared with the NDKD group, DKD patients had significantly longer diabetes duration, a higher prevalence of DR, higher SBP, and lower BMI (*P<*0.05). Additionally, patients with DKD exhibited significantly higher levels of FPG, HbA1c, BUN, serum RBP, serum cystatin C, urinary IgG, and urinary β2-MG, but lower levels of Hb, eGFR, total cholesterol, and LDL-C compared with the NDKD group (*P<*0.05). **Supplementary Table 4** displays the results of univariate logistic regression analysis of DKD risk factors in the training cohort.

Multivariable logistic regression analysis, which incorporated the significant variables from the univariate analysis, revealed that a longer duration of diabetes (*OR*: 1.012, 95% *CI*: 1.008–1.016, *P*<0.001), the presence of DR (*OR*: 5.416, 95% *CI*: 2.730–10.747, *P*<0.001), higher SBP (*OR*: 1.019, 95% *CI*: 1.003–1.037, *P=*0.023), higher FPG (*OR*: 1.327, 95% *CI*: 1.160–1.518, *P*<0.001), and lower Hb (*OR*: 0.969, 95% *CI*: 0.955–0.983, *P*<0.001) were independent risk factors for DKD (*P<*0.05). The VIFs for diabetes duration (1.028), DR (1.022), SBP (1.012), FPG (1.080), and Hb (1.060) ranged from 1.012 to 1.080. All VIFs were well below the commonly accepted threshold of 5, indicating no substantial multicollinearity among the predictors. Consequently, the multivariable regression model is unlikely to be meaningfully distorted by multicollinearity, and the estimated regression coefficients are therefore expected to be stable. **Table 3** displays the results of the multivariable logistic regression analysis of DKD risk factors in the training cohort.

**Table 3:** Multivariable logistic regression analysis of risk factors for DKD in patients with T2DM and renal impairment.

Variables	B	S.E.	Wald *χ*^2^	*OR* (95% *CI*)	*P* value
Diabetes duration (months)	0.012	0.002	30.011	1.012 (1.008, 1.016)	<0.001
Diabetic retinopathy (Yes/No)	1.689	0.350	23.349	5.416 (2.730, 10.747)	<0.001
SBP (mmHg)	0.019	0.008	5.183	1.019 (1.003, 1.037)	0.023
FPG (mmol/L)	0.283	0.069	17.026	1.327 (1.160, 1.518)	<0.001
Hb (g/L)	–0.031	0.007	18.974	0.969 (0.955, 0.983)	<0.001

B, coefficient; CI, confidence intervals; DKD, diabetic kidney disease; FPG, fasting plasma glucose; Hb, hemoglobin; OR, odds ratio; SBP, systolic blood pressure; S.E., standard error of the coefficient; T2DM, type 2 diabetes mellitus; Wald *χ*^2^, Wald chi-square statistic.

### 3.4 Model performance and internal validation

**Figure 2:**
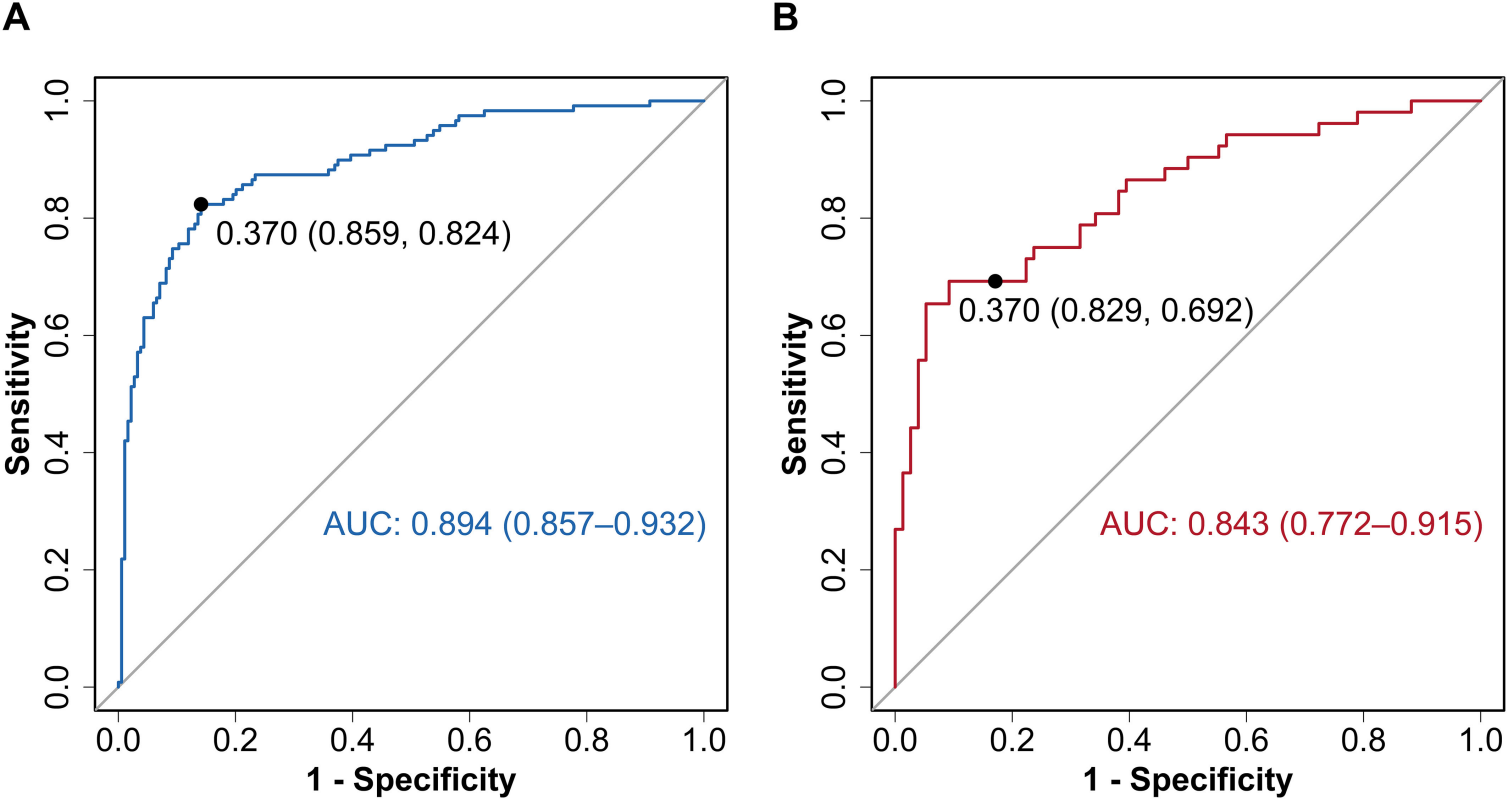
ROC curves of the diagnostic model for DKD. **(A)** ROC curve in the training cohort. **(B)** ROC curve in the validation cohort. AUC, area under the receiver operating characteristic curve; DKD, diabetic kidney disease; ROC, receiver operating characteristic.

**Figure 3:**
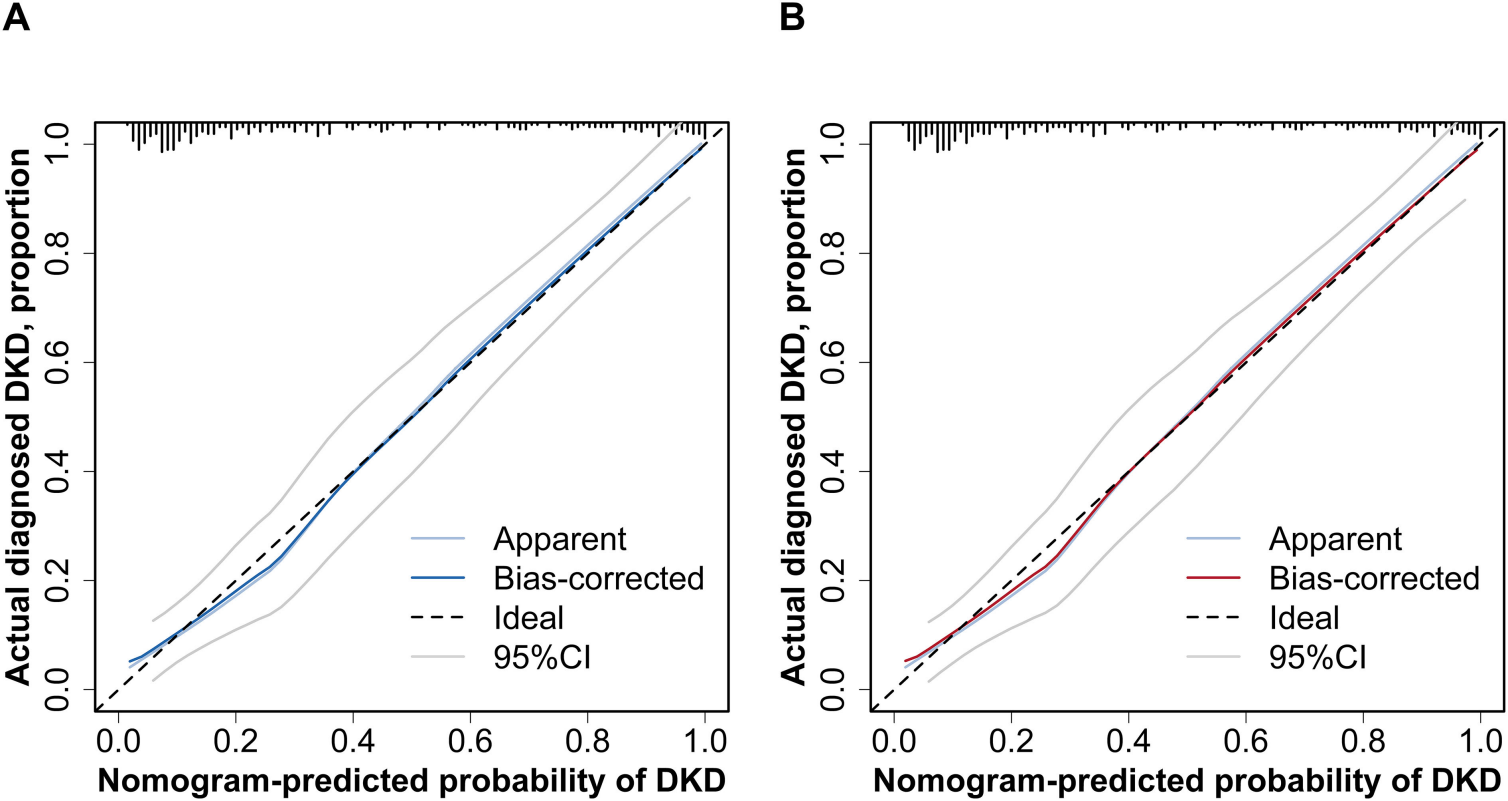
Calibration curves of the diagnostic model for DKD. **(A)** Calibration curve in the training cohort. **(B)** Calibration curve in the validation cohort. The calibration curves show satisfactory agreement with the ideal curve in both cohorts. “Apparent” represents the unadjusted calibration curve, showing the observed relationship between predicted probabilities and actual outcomes. “Bias-corrected” represents the bootstrap-adjusted calibration curve, which corrects for overfitting bias. “Ideal” represents the 45-degree reference line indicating perfect calibration. “95% CI” indicates the region between the two gray lines, representing the 95% confidence interval for the bias-corrected estimate. CI, confidence intervals; DKD, diabetic kidney disease.

**Figure 4:**
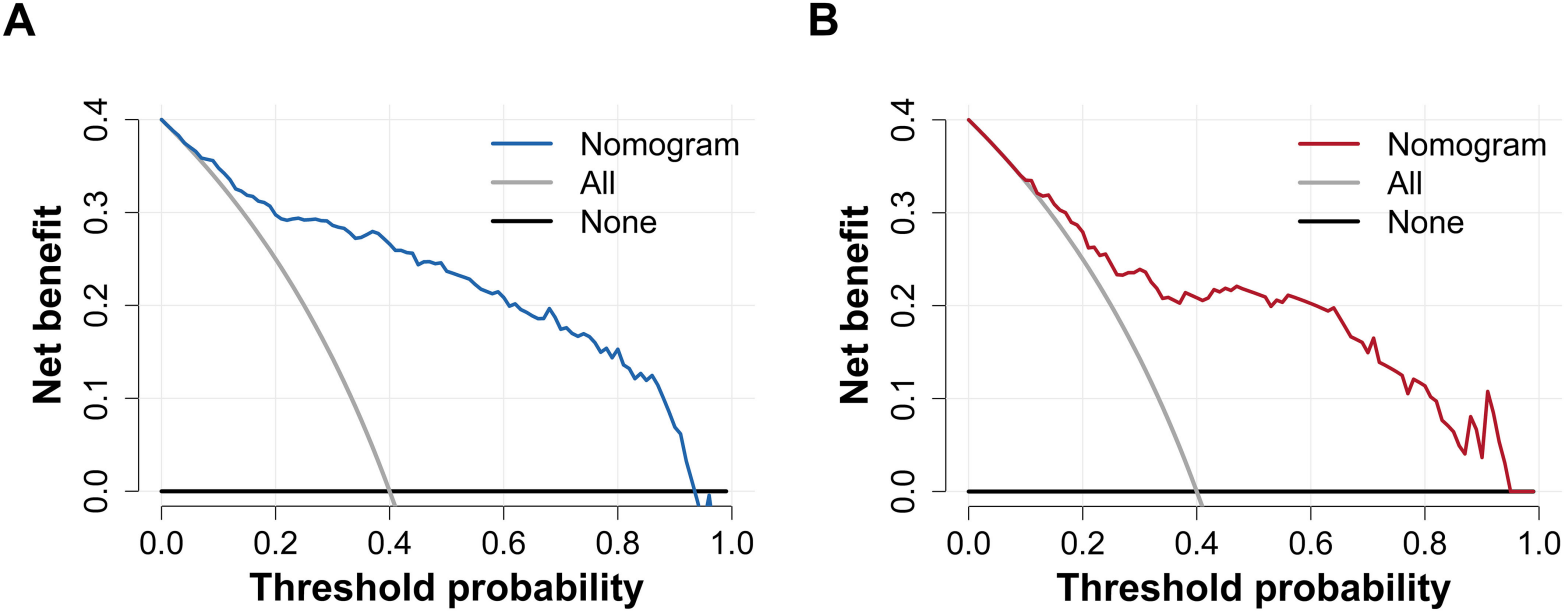
Clinical decision curves of the diagnostic model for DKD. **(A)** Clinical decision curve in the training cohort. **(B)** Clinical decision curve in the validation cohort. “Nomogram” indicates that the model provided a higher net benefit than either performing renal biopsy on all patients or performing no biopsy at all, with the threshold probability ranging from 0.05 to 0.93 in the training cohort and from 0.10 to 0.94 in the validation cohort. “All” indicates that the net benefit decreased as the threshold probability increased when renal biopsy was performed on all patients. “None” indicates that the net benefit was zero when renal biopsy was not performed on any patients. Abbreviations: DKD, diabetic kidney disease.

The final diagnostic model for DKD incorporated five key variables: diabetes duration, DR, SBP, FPG, and Hb. This diagnostic model exhibited excellent discriminatory performance. The *C*-statistics were 0.894 (95% *CI*: 0.857–0.932) and 0.843 (95% *CI*: 0.772–0.915) in the training and validation cohorts (**Figure 2**), and 0.774 (95% *CI*: 0.709–0.838) in the MIX group (**Supplementary Figure 4**). At the prespecified probability threshold of 0.370, the proportion of high-risk patients was similar between the training and validation cohorts (40.9% vs. 38.3%). Sensitivity, specificity, PPV, NPV, LR+, and LR– are provided in **Table 4**. The bias-corrected *C*-statistic derived from bootstrap resampling was 0.888 (95% *CI*: 0.853–0.928), with an estimated optimism of 0.006. The bootstrap-corrected calibration slope was 0.956, and the intercept was –0.017. The calibration curves showed satisfactory agreement with the ideal curve in both cohorts (**Figure 3**). The Hosmer-Lemeshow test demonstrated adequate model calibration in both the training cohort (*χ*²=0.561, degrees of freedom=2, *P=*0.755) and the validation cohort (*χ*²=5.375, degrees of freedom=2, *P=*0.068). Although the Hosmer-Lemeshow test yielded a *P* value of 0.016 (*χ*²=8.220, degrees of freedom=2) in the MIX group, calibration analysis demonstrated adequate agreement between the predicted and observed outcomes, indicating good model fit (**Supplementary Figure 4**). Clinical decision curve analysis showed that, in the training cohort, the model yielded a higher net benefit than either conducting renal biopsy on all patients or on none when the threshold probability ranged from 0.05 to 0.93. In the validation cohort, the model demonstrated a superior net benefit when the threshold probability ranged from 0.10 to 0.94. (**Figure 4**). In the MIX group, the model outperformed the strategies of performing renal biopsies on all patients or none, with a net benefit observed over the threshold probability range of 0.20 to 0.81 (**Supplementary Figure 4**).

**Table 4:** Diagnostic performance of the logistic regression model for DKD at the optimal probability threshold.

Group	AUC (95% *CI*)	Sensitivity (%)	Specificity (%)	PPV (%)	NPV (%)	Accuracy	LR+	LR–
Training cohort	0.894 (0.857–0.932)	82.4	85.9	79.0	88.3	0.845	5.83	0.21
Validation cohort	0.843 (0.772–0.915)	69.2	82.9	73.5	79.7	0.773	4.05	0.37
MIX group	0.774 (0.709–0.838)	57.9	85.0	53.0	87.4	0.789	3.86	0.50

The optimal probability threshold (0.370) was determined using the Youden index in the training cohort. AUC, area under the receiver operating characteristic curve; CI, confidence intervals; DKD, diabetic kidney disease; LR+, positive likelihood ratio; LR–, negative likelihood ratio; MIX group, NDKD and DKD combined with NDKD groups; NDKD, non-diabetic kidney disease; NPV, negative predictive value; PPV, positive predictive value.

### 3.5 Model presentation

**Figure 5:**
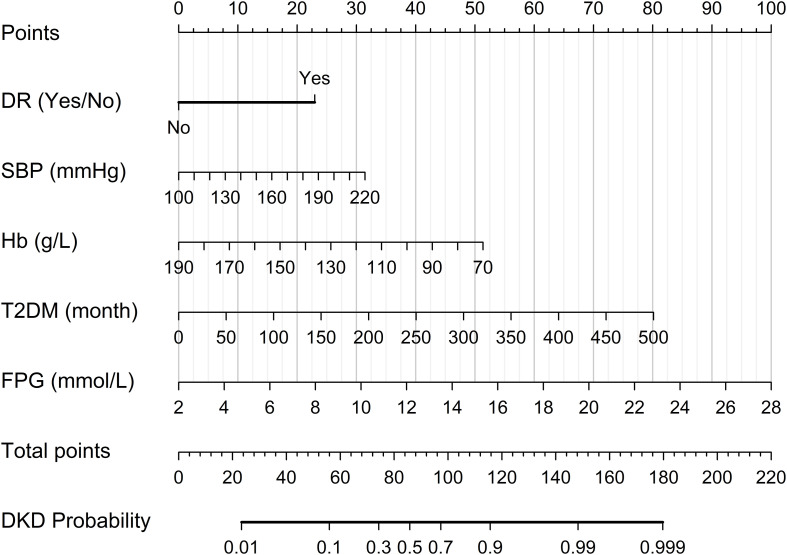
Nomogram of the diagnostic model for DKD in patients with T2DM and renal impairment. To use the nomogram: (1) For each variable, project its value onto the “Points” line to obtain an individual score; (2) Sum all individual scores to obtain the total score; (3) Locate the total score on the “Total points” line; (4) Project the total score downward to the “DKD Probability” line to estimate the risk of DKD in patients with T2DM and renal impairment. Abbreviations: DKD, diabetic kidney disease; DR, diabetic retinopathy; FPG, fasting plasma glucose; Hb, hemoglobin; SBP, systolic blood pressure; T2DM, type 2 diabetes mellitus.

Finally, the DKD diagnostic model was visualized as a nomogram (**Figure 5**). This nomogram enables the calculation of the probability of DKD by aggregating the scores assigned to five crucial clinical variables: duration of T2DM, DR, SBP, FPG, and Hb levels. It may provide a practical clinical tool for individual risk assessment in patients with T2DM and kidney injury.

## 4 Discussion

Renal impairment in patients with T2DM is a common clinical condition encompassing various types of renal diseases, such as DKD and NDKD. Notably, the course of DKD is heterogeneous, and awareness of DKD subtypes such as NADKD adds complexity to its clinical diagnosis (24). A composite diagnostic model based on the 2007 KDOQI criteria has been reported to demonstrate high sensitivity but limited specificity for distinguishing DKD from NDKD in a Chinese biopsy-proven cohort (sensitivity: 96.1%; specificity: 40.6%; PPV: 73.8%; NPV: 85.9%) (6). Therefore, a model that integrates a broader spectrum of variables is required to improve the accuracy of clinical differentiation between DKD and NDKD and reduce diagnostic bias. We developed and validated a diagnostic model that incorporates the duration of T2DM, DR, SBP, FPG, and Hb for patients with T2DM and renal injury. These variables can be readily obtained during routine clinical visits, and the nomogram may enable clinicians to identify patients with T2DM and renal impairment who are at high risk of developing DKD.

Initially, Zhou et al. developed a diagnostic model incorporating five risk factors (diabetes duration, diabetic retinopathy, HbA1c, systolic blood pressure, and microscopic hematuria), achieving a *C*-statistic of 0.968, with sensitivity and specificity of 90.0% and 92.0%, respectively (13). Liu et al. added Hb levels to a similar model, thereby improving its predictive performance and achieving a *C*-statistic of 0.971, with a sensitivity of 88.5% and a specificity of 91.0% (14). Some of these factors align with those used in our study. Our model achieved a *C*-statistic of 0.894 (95% *CI*: 0.857–0.932) in the training cohort and 0.843 (95% *CI*: 0.772–0.915) in the validation cohort. The bias-corrected *C*-statistic obtained via bootstrap resampling was 0.888 (95% *CI*: 0.853–0.928), with an estimated optimism of 0.006. The bootstrap-corrected calibration slope was 0.956, and the intercept was –0.017. These metrics collectively demonstrate that the model is robust and shows minimal evidence of overfitting. Additionally, we validated the model’s performance in the MIX group, where it yielded a *C*-statistic of 0.774 (95% *CI*: 0.709–0.838), with a specificity of 85.0% and a sensitivity of 57.9% at the cutoff value of 0.370. These results suggest that our model may also be applicable to patients with concurrent DKD and NDKD.

Although machine learning models have demonstrated promise, our nomogram provides a practical, user-friendly tool for clinical use (17). It could easily be adapted into a portable mobile application, thereby further streamlining clinical workflows. This nomogram is particularly valuable for patients with atypical presentations of diabetes-related kidney damage or for those who have contraindications to renal biopsy. By enabling risk stratification prior to invasive procedures, the model identifies patients at higher risk of developing DKD. This approach facilitates targeted interventions and helps avoid unnecessary renal biopsies, thereby minimizing potential harm. However, our model is intended as a decision-support tool, not as a definitive diagnostic tool. It should be used alongside clinical judgment and confirmatory tests, such as renal biopsy, especially when the diagnosis is uncertain. Relying solely on the model may lead to misclassification, potentially delaying appropriate treatment for patients with NDKD or subjecting those with DKD to unnecessary procedures, thereby compromising patient welfare.

In this study, we selected 0.370 as the clinical decision threshold to differentiate DKD from NDKD. At this threshold, the proportion of high-risk patients was similar between the training and validation cohorts (40.9% vs. 38.3%). And the model demonstrated high specificity in both cohorts (85.9% in the training cohort and 82.9% in the validation cohort), a feature critical to minimizing harmful misclassification, given the substantially different therapeutic pathways and prognoses between DKD and NDKD. In the training cohort, the LR+ was 5.83 and the LR– was 0.21 at this operating point, indicating clinically meaningful diagnostic utility. Additionally, decision curve analysis showed that, at a threshold probability of 0.370, the nomogram provided a higher net benefit than both the “all-biopsy” and “no-biopsy” strategies, further supporting its potential value in clinical triage.

Sustained inadequate glycemic management significantly increases the risk of DKD development. The KDOQI guidelines recommend initiating DKD screening at the time of T2DM diagnosis (5). The correlation between hyperglycemia and DKD has been well established, with hyperglycemia playing a central role in the initiation of DKD (25). Longer duration of T2DM is consistently associated with a higher risk of complications, a pattern that aligns with epidemiological observations in Pima Indians (26). Stricter glycemic control was associated with a lower incidence of microvascular complications among individuals with T2DM (27). Furthermore, the beneficial effect of intensive glucose-lowering therapy persisted, even though the differences in mean HbA1c levels and therapeutic approaches between the intensive and conventional glycemic treatment groups were no longer statistically significant—a phenomenon that can be explained by the concept of “metabolic memory” (28). These findings underscore the crucial importance of optimal glycemic control in preventing DKD.

DR, a classic diagnostic marker for DKD, frequently coexists with early-stage DKD (29, 30). Similar pathological alterations have been detected in the glomerular and retinal vasculature of patients with diabetes (31, 32). DR is independently associated with renal functional deterioration. Diabetic patients with comorbid DR had a 2.6-fold higher risk of experiencing a serum creatinine increase of ≥35.4 μmol/L, hospitalization, or death due to kidney disease within 6 years compared to those without DR (31). A meta-analysis has confirmed that DR is a highly effective predictor for differentiating DKD from NDKD (33). These findings highlight the clinical significance of fundus examination as a straightforward and non-invasive approach for the early detection of DKD. Nevertheless, it is important to note that NDKD cannot be excluded in T2DM patients with a disease duration of over 10 years or the presence of DR (34). Furthermore, T2DM patients with renal impairment who do not exhibit DR may still develop DKD. As demonstrated in our study, among the 431 patients in the DKD and NDKD groups, 24.32% of those without DR were diagnosed with biopsy-confirmed DKD. Relying solely on DR to differentiate DKD from NDKD may lead to incorrect classification. This underscores the importance of integrating additional clinical variables, such as SBP, Hb, and FPG, to enhance diagnostic accuracy and reduce the risk of missed diagnoses.

We found that a higher SBP level at admission was more strongly indicative of DKD. DKD and hypertension exhibit a bidirectional, mutually reinforcing relationship. This may be ascribed to the activation of the renin-angiotensin-aldosterone system (RAAS) and to the enhanced vasoconstrictive effects of endothelin in patients with DKD (35). Observational analyses of the United Kingdom Prospective Diabetes Study (UKPDS) cohort showed a strong association between SBP levels and the onset of DKD in T2DM patients (36). Specifically, each 10-mmHg decline in mean SBP level was associated with a 13% decrease in the risk of developing diabetic microvascular complications (36). A 7-year longitudinal analysis of the Rio de Janeiro T2DM Cohort identified higher mean daytime ambulatory SBP as a significant risk factor for renal function decline (37). These findings indicate that stable and effective blood pressure management is crucial for preventing diabetes-related microvascular complications. Therefore, incorporating SBP levels in our model may enhance the accuracy of clinical diagnosis for DKD.

It is worth noting that Hb levels offered novel discriminatory value. Patients with DKD had significantly lower Hb levels than those with NDKD. Previous research showed that anemia developed earlier and was more severe in patients with DKD than in those with NDKD (38, 39). In the Kidney Early Evaluation Program (KEEP) 2.0 cohort study, the prevalence of anemia was significantly higher in patients with DKD than in those with NDKD at CKD stage 3 (40). Notably, a 5-year prospective study illustrating the natural progression of Hb levels in patients with T2DM demonstrated that a decrease in Hb levels can occur even at an early stage of DKD (41). In DKD, early erythropoietin dysfunction—driven by hyperglycemia-induced tubulointerstitial hypoxia, sympathetic denervation, and chronic inflammation—plays a central role in the development of anemia. Notably, this erythropoietin deficiency occurs even before significant declines in GFR or the onset of overt albuminuria. In contrast, anemia in NDKD is more directly linked to nephron loss and progressive decline in renal function. In NDKD, erythropoietin deficiency typically becomes significant when the disease progresses to end-stage kidney disease. These distinct pathophysiological mechanisms underlying anemia in DKD and NDKD underscore the role of Hb levels as an early and sensitive marker for DKD progression (42). This highlights the importance of regular anemia screening for the early detection of diabetes-related microvascular complications. When patients with T2DM and renal impairment exhibit lower Hb levels, clinicians should suspect DKD.

However, this study has several limitations. First, the single-center design may introduce selection bias, limiting the generalizability of our findings. Second, by exclusively including T2DM patients with renal impairment who underwent renal biopsy, the study may introduce selection bias, thereby limiting the model’s applicability to patients with less severe kidney disease or those who have not undergone biopsy. Moreover, although DKD was used as the outcome measure, subgroup analysis by DKD grade was not performed, which represents another limitation. Additionally, the model may carry a risk of missed diagnoses among DKD patients without DR. Third, the retrospective case-control design limits our ability to establish causal associations between the identified risk factors and renal damage. Although multiple covariates were adjusted for, the possibility of residual confounding (such as medication use and socioeconomic factors) cannot be entirely ruled out, potentially affecting the interpretation of the findings. Fourth, the optimal threshold may vary with local disease prevalence, biopsy practices, and the relative harms of false-positive and false-negative classifications. Finally, the lack of follow-up data on patient outcomes limits our ability to assess long-term prognosis. Therefore, we acknowledge the need for multi-center prospective validation across diverse populations to confirm the broader applicability and external validity of our findings.

In conclusion, we developed and validated a comprehensive diagnostic model incorporating diabetes duration, DR, SBP, FPG, and Hb, which enables effective differentiation between DKD and NDKD in patients with T2DM and renal impairment. Hb may serve as a promising predictor for DKD diagnosis beyond traditional parameters. The nomogram format may simplify clinical implementation and facilitate quick assessment during routine consultations.

## Data Availability

The original contributions presented in the study are included in the article/**Supplementary Material**. Further inquiries can be directed to the corresponding author.

## Supplementary material

Supplementary Table 1 Stability of multivariable logistic regression coefficients across five imputed datasets.

Supplementary Table 2 Model performance at the optimal probability threshold across five imputed datasets.

Supplementary Table 3 Baseline characteristics of the training and validation cohorts.

Supplementary Table 4 Univariate logistic regression analysis of risk factors for DKD in patients with T2DM and renal impairment.

Supplementary Figure 1 Flowchart of patient screening.

Supplementary Figure 2 Variable-specific missingness proportions.

Supplementary Figure 3 Intersection of missing data patterns.

Supplementary Figure 4 Model performance in the MIX group.

## Glossary

AUC: area under the receiver operating characteristic curve
β2-MG: β2-microglobulin
BMI: body mass index
BUN: blood urea nitrogen
CI: confidence intervals
CKD: chronic kidney disease
CKD-EPI: Chronic Kidney Disease Epidemiology Collaboration
DALYs: disability-adjusted life-years
DBP: diastolic blood pressure
DKD: diabetic kidney disease
DR: diabetic retinopathy
eGFR: estimated glomerular filtration rate
ESR: erythrocyte sedimentation rate
FPG: fasting plasma glucose
Hb: hemoglobin
HbA1c: glycated hemoglobin
HDL-C: high-density lipoprotein cholesterol
Hs-CRP: high-sensitivity C-reactive protein
IgG: immunoglobulin G
IQR: interquartile range
KDOQI: Kidney Disease Outcomes Quality Initiative
KEEP: Kidney Early Evaluation Program
LDL-C: low-density lipoprotein cholesterol
LR+: positive likelihood ratio
LR–: negative likelihood ratio
MCAR: missing completely at random
MICE: multiple imputation by chained equations
MIX group: NDKD and DKD combined with NDKD groups
NADKD: normoalbuminuric diabetic kidney disease
NDKD: non-diabetic kidney disease
NPV: negative predictive value
OR: odds ratio
PPV: positive predictive value
RAAS: renin-angiotensin-aldosterone system
RBP: retinol-binding protein
ROC: receiver operating characteristic
SBP: systolic blood pressure
T2DM: type 2 diabetes mellitus
TRIPOD: Transparent Reporting of a multivariable prediction model for Individual Prognosis Or Diagnosis
UAE: urinary albumin excretion
UKPDS: United Kingdom Prospective Diabetes Study
UPE: urinary protein excretion
VIF: variance inflation factor
WHO: World Health Organization.
