# Supplementary material for: Development and validation of a multivariable prediction model for non-invasive discrimination between diabetic and non-diabetic kidney disease in type 2 diabetes: a clinical nomogram
Source: Front Endocrinol (Lausanne). 2026 Mar 5;17:1787412. doi: 10.3389/fendo.2026.1787412 (PMC12999389; doi:10.3389/fendo.2026.1787412)
Supplement: Supplementary Table 1 — Stability of multivariable logistic regression coefficients across five imputed datasets. [file DataSheet1.docx]

Supplementary Material

# Patient Screening

A total of 2811 patients were screened. Among them, 2671 were aged 18 years or older, and 560 had type 2 diabetes. The exclusion procedures were as follows: 14 patients were excluded due to incomplete data, and 39 were excluded because of a history of renal transplantation, urinary tract infection, urolithiasis, or urological malignancy. Ultimately, 507 patients with type 2 diabetes and renal impairment were included in the final analysis. The flow from the initial cohort to the analytic cohort is illustrated in **Supplementary Figure 1**.

# Missing Data Pattern

Among the 507 participants, 349 (68.8%) had complete data across all 33 variables. Among the remaining cases, 31 distinct missing data patterns were identified. The missingness rate for individual variables ranged from 0.2% to 13.2%; the most frequently missing variables were ESR (13.2%), HbA1c (9.5%), cystatin C (9.1%), and RBP (8.9%), whereas all other variables had missing rates below 5% (**Supplementary Figure 2**). We assessed the missing-data mechanism using Little’s Missing Completely at Random (MCAR) test ($\chi$^2^$=$793.0, degrees of freedom$=$742, *P*$=$0.095), which did not provide evidence against the MCAR assumption. This supports the use of imputation to reduce potential bias, preserve sample size, and maintain statistical power. Detailed missingness patterns are visualized in **Supplementary Figures 3**.

# Missing Data Handling

Missing values were addressed prior to model construction. Variables with missing values were imputed using Multiple Imputation by Chained Equations (MICE) via the “mice” package in R. Specifically, Predictive Mean Matching (PMM) was applied for continuous variables, and logistic regression was used for binary categorical variables. Five imputed datasets were generated, and the first complete dataset was selected for the primary analysis. Sensitivity analyses conducted across all five imputed datasets yielded identical coefficient estimates (**Supplementary Table 1**), demonstrating that the model was robust to the imputation of auxiliary variables. This exceptional stability is attributable to the minimal amount of missingness in the final predictor variables. Furthermore, discriminative performance remained highly consistent, with the *C*-statistic stable at 0.894 (95% *CI*: 0.857–0.932) across all imputed datasets (**Supplementary Table 2**). Consequently, results from the first imputed dataset are presented as representative. These results confirm that the imputation process did not introduce bias and that the reported clinical inferences are statistically robust.

# Supplementary Figures and Tables

## Supplementary Figures


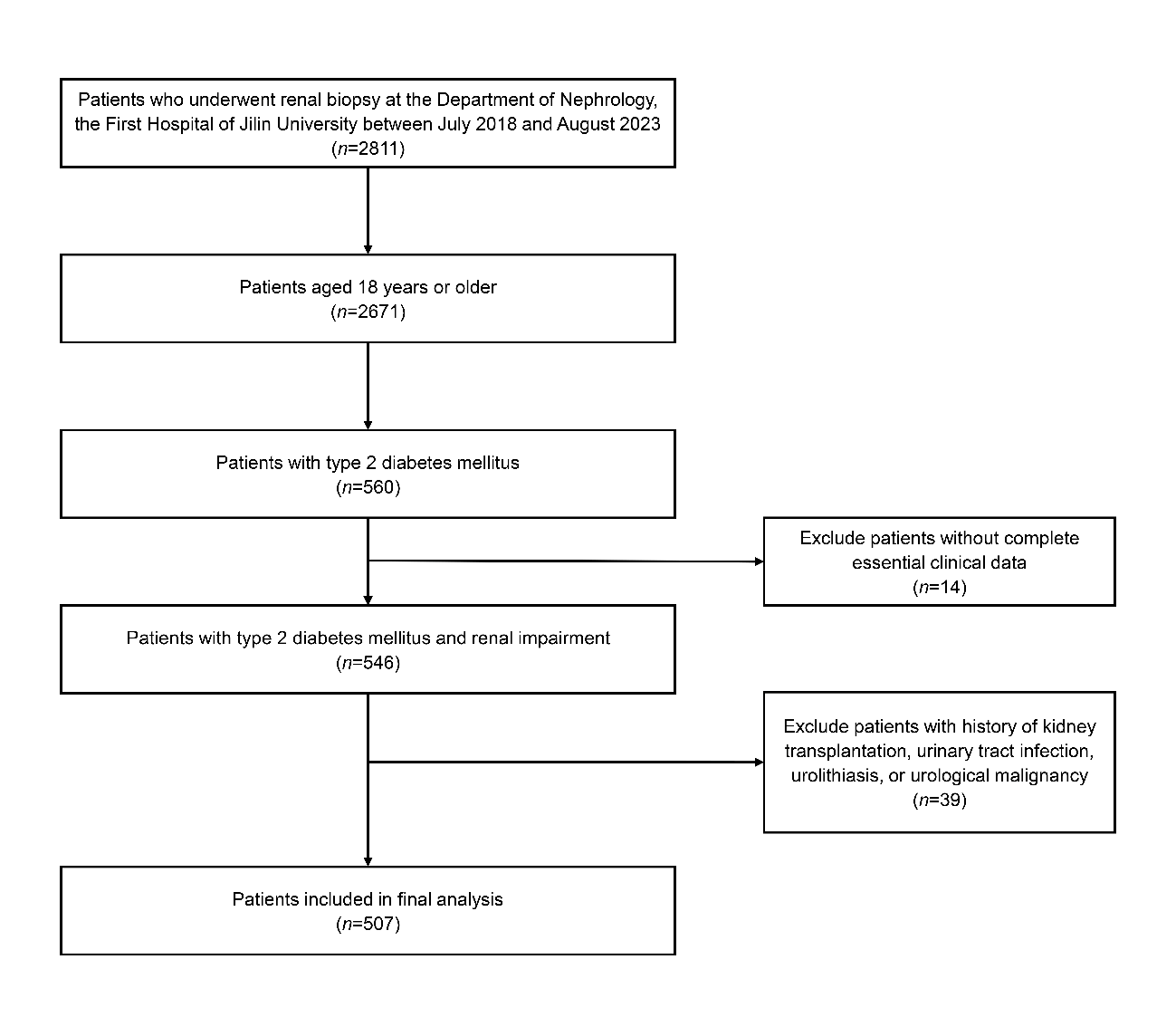


**Supplementary Figure 1.** Flowchart of patient screening.


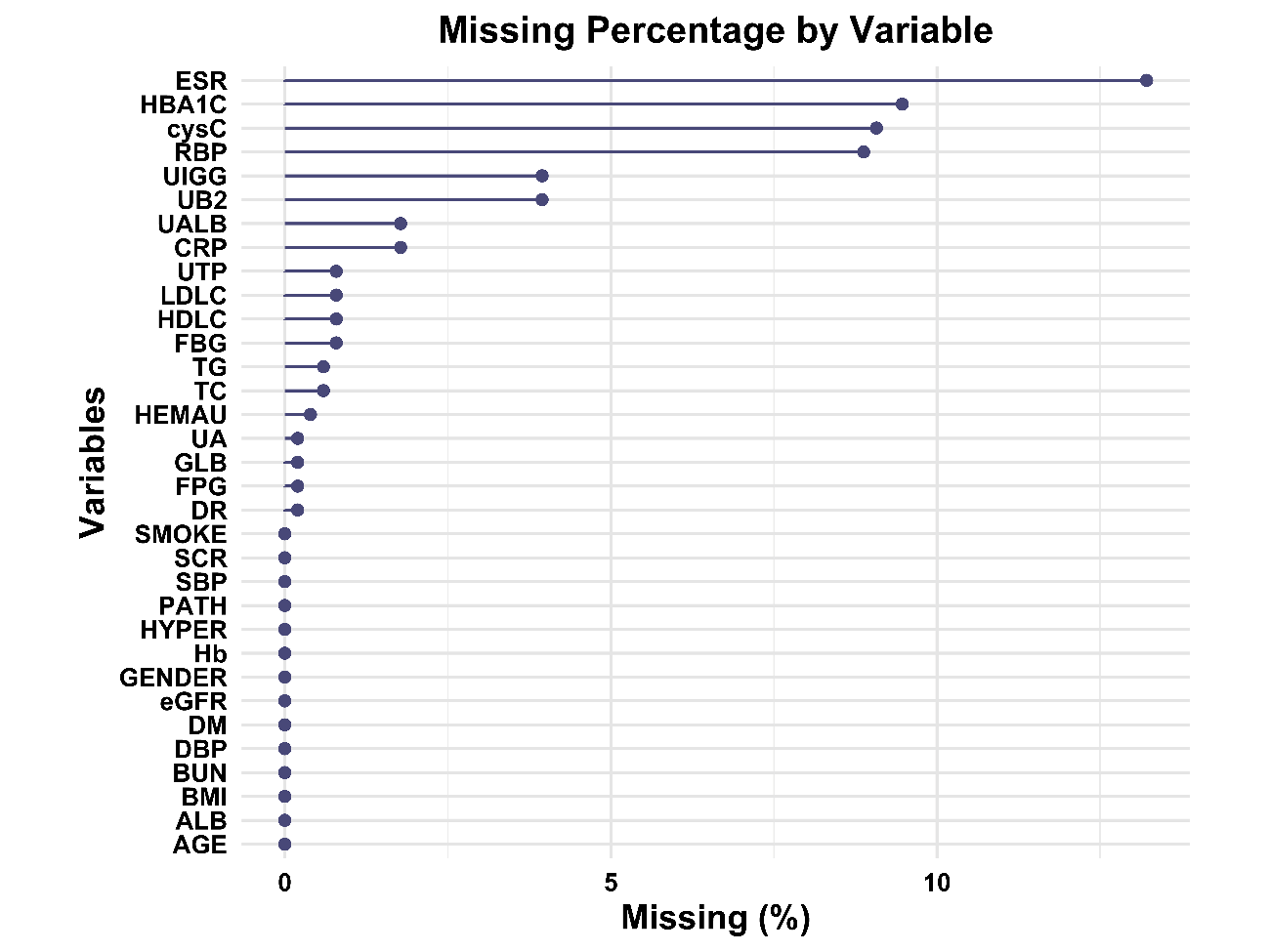


**Supplementary Figure 2.** Variable-specific missingness proportions. Proportional distribution of missing values across the 33 clinical variables. The bars are arranged in descending order of missingness frequency. ESR exhibited the highest missingness rate (13.2%), followed by HbA1c (9.5%). Most variables demonstrated high data integrity, with missingness rates below 5%. Abbreviations: ALB, serum albumin; BMI, body mass index; BUN, blood urea nitrogen; CRP, high-sensitivity C-reactive protein; cysC, cystatin C; DBP, diastolic blood pressure; DM, diabetes duration; DR, diabetic retinopathy; eGFR, estimated glomerular filtration rate; ESR, erythrocyte sedimentation rate; FBG, fibrinogen; FPG, fasting plasma glucose; GLB, serum globulin; HbA1c, glycated hemoglobin; HDLC, high-density lipoprotein cholesterol; HEMAU, microscopic hematuria; HYPER, hypertension; Hb, hemoglobin; LDLC, low-density lipoprotein cholesterol; PATH, renal pathology; RBP, retinol-binding protein; SBP, systolic blood pressure; SCR, serum creatinine; TC, total cholesterol; TG, triglycerides; UALB, 24-hour urinary albumin excretion; UB2, $\beta$2-microglobulin; UIGG, urinary immunoglobulin G; UTP, 24-hour urinary protein excretion.


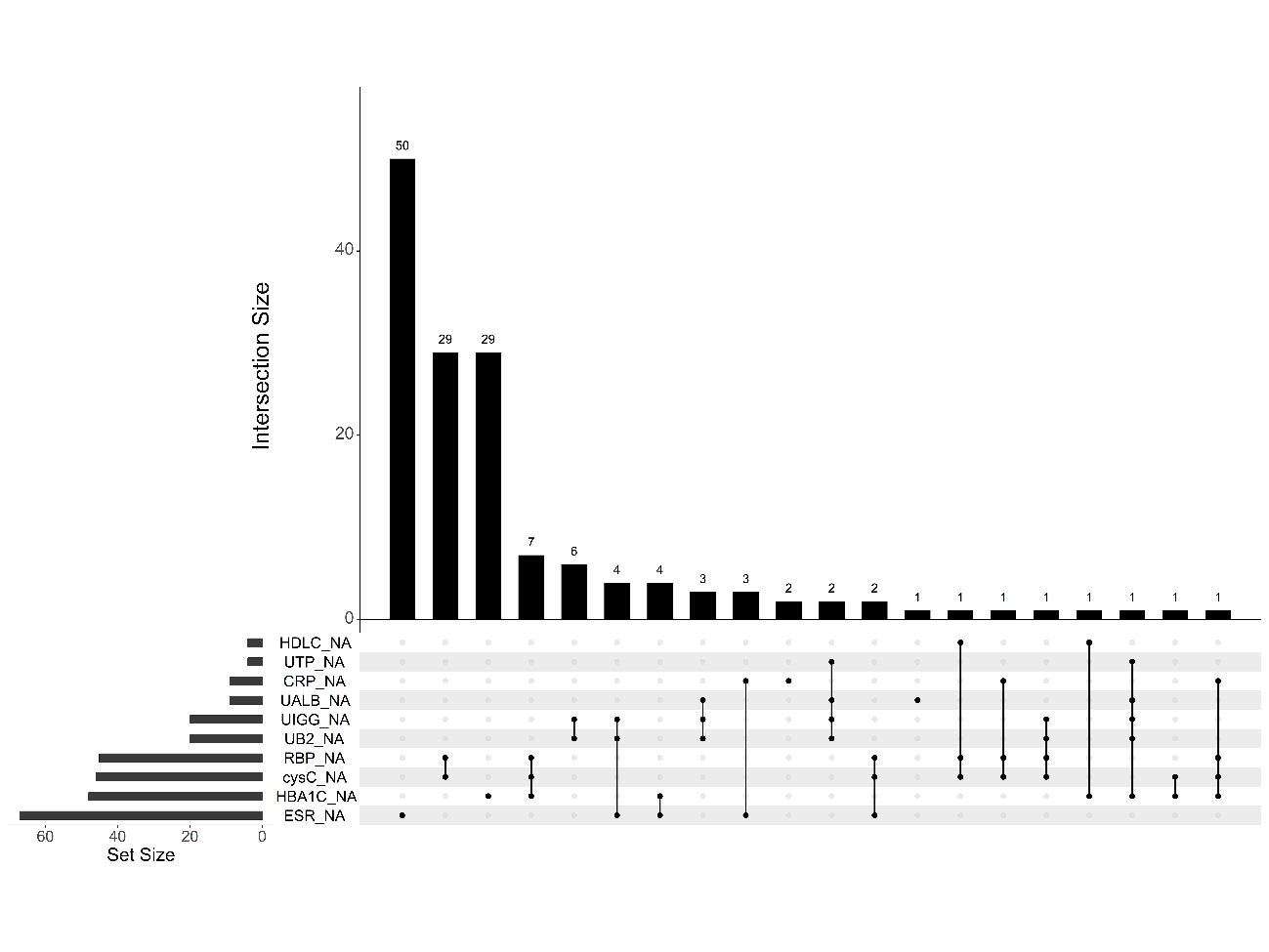


**Supplementary Figure 3.** Intersection of missing data patterns. An UpSet plot illustrating the 31 distinct missing data patterns identified in the cohort. The vertical bars represent the frequency of each specific missingness intersections (i.e., combinations), and the matrix below highlights the variables involved. NA represents the missing values for each variable. The visualization reveals that the majority of missing data occur as singleton patterns (e.g., cases missing only ESR), with few instances of complex co-missingness across multiple parameters, consistent with a MCAR mechanism. Abbreviations: CRP, high-sensitivity C-reactive protein; cysC, cystatin C; ESR, erythrocyte sedimentation rate; HbA1c, glycated hemoglobin; HDLC, high-density lipoprotein cholesterol; MCAR, Missing Completely at Random; RBP, retinol-binding protein; UALB, 24-hour urinary albumin excretion; UB2, $\beta$2-microglobulin; UIGG, urinary immunoglobulin G; UTP, 24-hour urinary protein excretion.


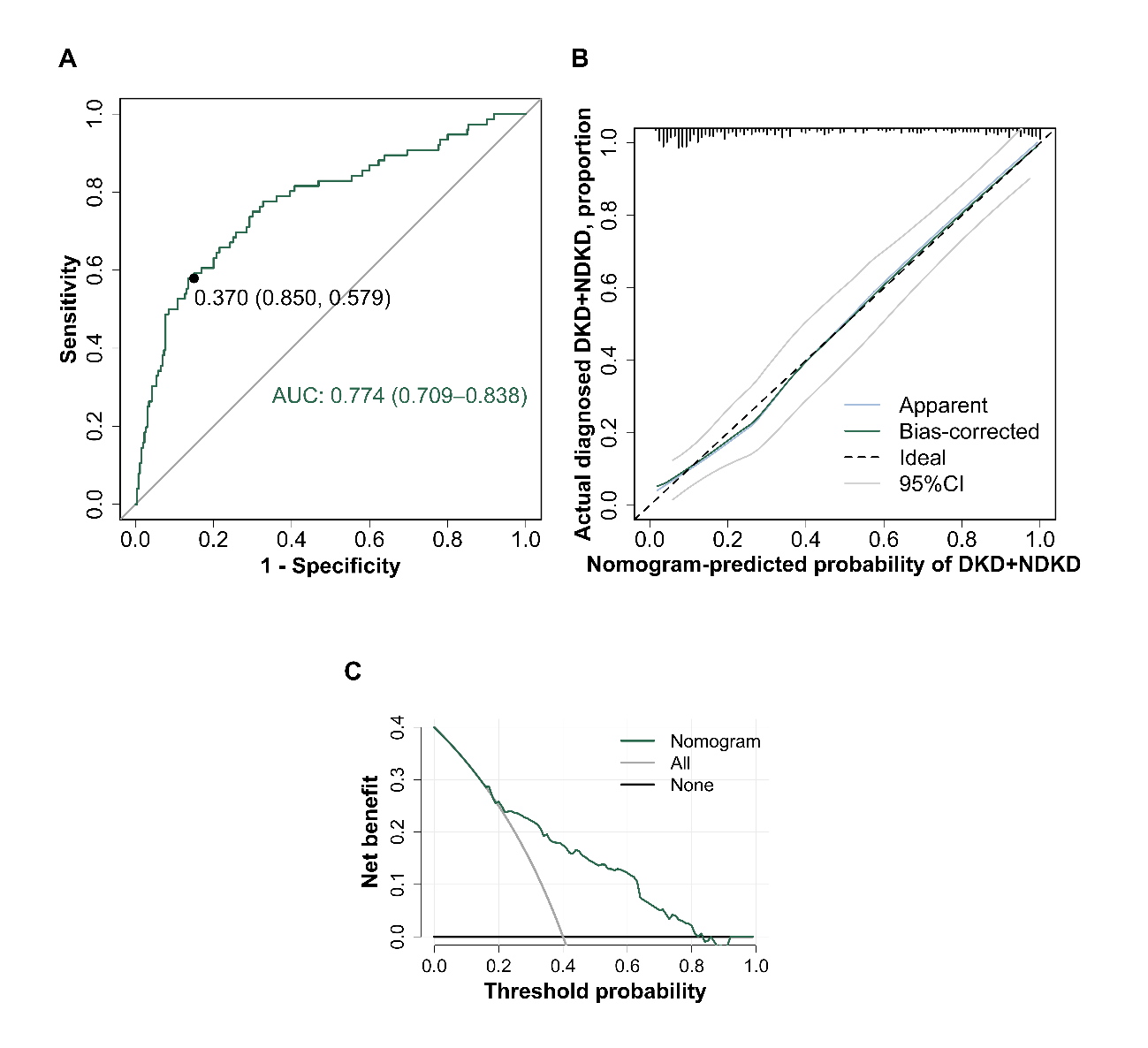


**Supplementary Figure 4.** Model performance in the MIX group. **(A)** ROC curve of the model in the MIX group. **(B)** Calibration curve of the model in the MIX group. The calibration curve shows good agreement with the ideal curve in the MIX group. “Apparent” represents the unadjusted calibration curve, showing the observed relationship between predicted probabilities and actual outcomes. “Bias-corrected” represents the bootstrap-adjusted calibration curve, which corrects for overfitting bias. “Ideal” represents the 45-degree reference line, indicating perfect calibration. “95% CI” indicates the region between the two gray lines, representing the 95% confidence interval for the bias-corrected estimate. (C) Clinical decision curve of the model in the MIX group. “Nomogram” indicates that the model provides a higher net benefit than either performing renal biopsy on all patients or performing no biopsy, across the threshold probability range of 0.20 to 0.81 in the MIX group. “All” indicates that the net benefit decreases as the threshold probability increases when renal biopsy is performed on all patients. “None” indicates that the net benefit is zero when no renal biopsy is performed. Abbreviations: AUC, area under the receiver operating characteristic curve; CI, confidence intervals; DKD, diabetic kidney disease; DKD+NDKD, DKD combined with NDKD; MIX group: NDKD and DKD combined with NDKD groups; NDKD, non-diabetic kidney disease; ROC, receiver operating characteristic.

## Supplementary Tables

**Supplementary Table 1. Stability of multivariable logistic regression coefficients across five imputed datasets**

| Variables | Dataset 1 | Dataset 2 | Dataset 3 | Dataset 4 | Dataset 5 | Mean | SD |
| --- | --- | --- | --- | --- | --- | --- | --- |
| Intercept | $-$2.8227 | $-$2.8227 | $-$2.8227 | $-$2.8227 | $-$2.8227 | $-$2.8227 | $<$0.001 |
| Diabetes duration (months) | 0.0118 | 0.0118 | 0.0118 | 0.0118 | 0.0118 | 0.0118 | $<$0.001 |
| Diabetic retinopathy (Yes/No) | 1.6894 | 1.6894 | 1.6894 | 1.6894 | 1.6894 | 1.6894 | $<$0.001 |
| SBP (mmHg) | 0.0193 | 0.0193 | 0.0193 | 0.0193 | 0.0193 | 0.0193 | $<$0.001 |
| FPG (mmol/L) | 0.2829 | 0.2829 | 0.2829 | 0.2829 | 0.2829 | 0.2829 | $<$0.001 |
| Hb (g/L) | $-$0.0315 | $-$0.0315 | $-$0.0315 | $-$0.0315 | $-$0.0315 | $-$0.0315 | $<$0.001 |

The values represent the regression coefficients derived from each of the five imputed datasets, along with their mean and variability. Notably, the coefficients were highly consistent across all datasets (between-imputation standard deviation$<$0.001), reflecting the minimal extent of missingness in these predictors. Abbreviations: FPG, fasting plasma glucose; Hb, hemoglobin; SBP, systolic blood pressure; SD, standard deviation.

**Supplementary Table 2. Model performance at the optimal probability threshold across five imputed datasets**

| Datasets | AUC (95% *CI*) | Sensitivity (%) | Specificity (%) | PPV (%) | NPV (%) | Accuracy | LR$+$ | LR$-$ |
| --- | --- | --- | --- | --- | --- | --- | --- | --- |
| Dataset 1 | 0.894 (0.857–0.932) | 82.4 | 85.9 | 79.0 | 88.3 | 0.845 | 5.83 | 0.21 |
| Dataset 2 | 0.894 (0.857–0.932) | 82.4 | 85.9 | 79.0 | 88.3 | 0.845 | 5.83 | 0.21 |
| Dataset 3 | 0.894 (0.857–0.932) | 82.4 | 85.9 | 79.0 | 88.3 | 0.845 | 5.83 | 0.21 |
| Dataset 4 | 0.894 (0.857–0.932) | 82.4 | 85.9 | 79.0 | 88.3 | 0.845 | 5.83 | 0.21 |
| Dataset 5 | 0.894 (0.857–0.932) | 82.4 | 85.9 | 79.0 | 88.3 | 0.845 | 5.83 | 0.21 |

Model performance metrics were evaluated across five imputed datasets using a fixed probability threshold of 0.370. The performance indices were consistent across all datasets, underscoring the robustness of the model’s predictive accuracy. This stability was maintained despite the imputation of minor missing values. Abbreviations: AUC, area under the receiver operating characteristic curve; CI, confidence interval; LR$+$, positive likelihood ratio; LR$-$, negative likelihood ratio; NPV, negative predictive value; PPV, positive predictive value.

**Supplementary Table 3. Baseline characteristics of the training and validation cohorts**

| Characteristics | Training cohort (*n*$=$303) | Validation cohort (*n*$=$128) | *P* value |
| --- | --- | --- | --- |
| Male | 183 (60.4) | 76 (59.4) | 0.928 |
| Age (years) | 54 (44, 61) | 54 (44, 61) | 0.635 |
| BMI (kg/m^2^) | 25.8 (23.4, 28.4) | 25.7 (24.0, 28.2) | 0.826 |
| Diabetes duration (months) | 48 (3, 120) | 48 (6, 120) | 0.952 |
| Diabetic retinopathy | 95 (31.4) | 40 (31.2) | 1.000 |
| Hypertension duration (months) | 12 (0, 72) | 12 (0, 60) | 0.934 |
| SBP (mmHg) | 140 (130, 155) | 145 (132, 158) | 0.124 |
| DBP (mmHg) | 82 (75, 90) | 83 (75, 90) | 0.754 |
| Smoker | 60 (19.8) | 30 (23.4) | 0.472 |
| FPG (mmol/L) | 6.37 (5.31, 7.74) | 6.39 (5.17, 7.84) | 0.863 |
| HbA1c (%) | 7.0 (6.2, 7.9) | 7.0 (6.2, 8.5) | 0.542 |
| Hb (g/L) | 129 (108, 145) | 132 (108, 143) | 0.374 |
| Hs-CRP (mg/L) | 3.02 (1.48, 5.53) | 3.02 (1.69, 4.79) | 0.530 |
| ESR (mm/h) | 42 (24, 64) | 39 (25, 58) | 0.819 |
| Fibrinogen (g/L) | 4.28 (3.40, 5.64) | 4.13 (3.52, 5.02) | 0.242 |
| Serum albumin (g/L) | 29.9 (23.5, 35.5) | 30.9 (24.9, 36.2) | 0.200 |
| Serum globulin (g/L) | 26.0 (23.3, 29.3) | 26.2 (23.2, 29.8) | 0.491 |
| BUN (mmol/L) | 7.08 (5.36, 9.91) | 7.09 (5.59, 10.25) | 0.592 |
| Serum creatinine ($\mu$mol/L) | 101.9 (72.3, 157.4) | 97.2 (76.7, 159.2) | 0.817 |
| Serum RBP (mg/L) | 59.5 (47.3, 79.3) | 64.4 (50.4, 78.5) | 0.439 |
| Serum cystatin C (mg/L) | 1.35 (1.00, 2.03) | 1.31 (1.01, 2.00) | 0.751 |
| Serum uric acid ($\mu$mol/L) | 393 (332, 461) | 412 (318, 484) | 0.371 |
| eGFR (mL/min/1.73m^2^) | 63.00 (38.00, 94.50) | 70.50 (39.00, 90.00) | 0.829 |
| Triglycerides (mmol/L) | 2.16 (1.45, 3.44) | 2.28 (1.51, 3.29) | 0.689 |
| Total cholesterol (mmol/L) | 5.58 (4.68, 7.50) | 6.02 (4.85, 7.55) | 0.514 |
| LDL-C (mmol/L) | 3.57 (2.88, 4.64) | 3.54 (2.88, 4.69) | 0.823 |
| HDL-C (mmol/L) | 1.20 (0.98, 1.48) | 1.20 (1.03, 1.51) | 0.483 |
| Microscopic hematuria | 166 (54.8) | 75 (58.6) | 0.534 |
| UPE (g/24h) | 3.860 (1.790, 7.590) | 3.640 (1.680, 7.310) | 0.635 |
| UAE (mg/24h) | 2927.00 (1270.77, 5572.36) | 2830.27 (1161.72, 5212.88) | 0.618 |
| Urinary IgG (mg/24h) | 247.34 (93.90, 613.92) | 274.01 (79.93, 602.04) | 0.880 |
| Urinary $\beta$2-MG (mg/24h) | 0.89 (0.19, 10.85) | 1.33 (0.19, 7.70) | 0.509 |
| DKD | 119 (39.3) | 52 (40.6) | 0.877 |

Data are presented as median (IQR) for skewed continuous variables and as n (%) for categorical variables. Mann-Whitney *U* tests for non-normally distributed continuous variables, and chi-square tests for categorical variables. Abbreviations: $\beta$2-MG, $\beta$2-microglobulin; BMI, body mass index; BUN, blood urea nitrogen; DBP, diastolic blood pressure; DKD, diabetic kidney disease; eGFR, estimated glomerular filtration rate; ESR, erythrocyte sedimentation rate; FPG, fasting plasma glucose; Hb, hemoglobin; HbA1c, glycated hemoglobin; HDL-C, high-density lipoprotein cholesterol; Hs-CRP, high-sensitivity C-reactive protein; IgG, immunoglobulin G; IQR, interquartile range; LDL-C, low-density lipoprotein cholesterol; RBP, retinol-binding protein; SBP, systolic blood pressure; UAE, urinary albumin excretion; UPE, urinary protein excretion.

**Supplementary Table 4. Univariate logistic regression analysis of risk factors for DKD in patients with T2DM and renal impairment**

| Variables | B | S.E. | Wald $\chi$^2^ | *OR* (95% *CI*) | *P* value |
| --- | --- | --- | --- | --- | --- |
| Sex (Male/Female) | -0.281 | 0.240 | 1.370 | 0.755 (0.472, 1.208) | 0.242 |
| Age (years) | -0.005 | 0.010 | 0.248 | 0.995 (0.975, 1.015) | 0.618 |
| BMI (kg/m^2^) | -0.066 | 0.031 | 4.630 | 0.936 (0.881, 0.994) | 0.031$\dagger$ |
| Diabetes duration (months) | 0.013 | 0.002 | 53.075 | 1.014 (1.010, 1.017) | $<$0.001$\dagger$ |
| Diabetic retinopathy (Yes/No) | 2.207 | 0.285 | 60.130 | 9.086 (5.201, 15.870) | $<$0.001$\dagger$ |
| Hypertension duration (months) | -0.001 | 0.002 | 0.436 | 0.999 (0.996, 1.002) | 0.509 |
| SBP (mmHg) | 0.024 | 0.006 | 13.989 | 1.024 (1.011, 1.037) | $<$0.001$\dagger$ |
| DBP (mmHg) | 0.000 | 0.010 | 0.000 | 1.000 (0.980, 1.020) | 0.994 |
| Smoker (Yes/No) | -0.138 | 0.298 | 0.213 | 0.871 (0.486, 1.563) | 0.644 |
| FPG (mmol/L) | 0.189 | 0.047 | 16.420 | 1.208 (1.102, 1.323) | $<$0.001$\dagger$ |
| HbA1c (%) | 0.225 | 0.074 | 9.145 | 1.252 (1.082, 1.449) | 0.002$\dagger$ |
| Hb (g/L) | -0.036 | 0.006 | 39.248 | 0.965 (0.954, 0.976) | $<$0.001$\dagger$ |
| Hs-CRP (mg/L) | -0.003 | 0.008 | 0.091 | 0.997 (0.981, 1.014) | 0.763 |
| ESR (mm/h) | 0.006 | 0.004 | 2.213 | 1.006 (0.998, 1.013) | 0.137 |
| Fibrinogen (g/L) | 0.029 | 0.076 | 0.146 | 1.029 (0.887, 1.194) | 0.702 |
| Serum albumin (g/L) | 0.011 | 0.015 | 0.557 | 1.011 (0.982, 1.041) | 0.455 |
| Serum globulin (g/L) | 0.003 | 0.024 | 0.020 | 1.003 (0.957, 1.052) | 0.887 |
| BUN (mmol/L) | 0.091 | 0.028 | 10.336 | 1.095 (1.036, 1.157) | 0.001$\dagger$ |
| Serum creatinine ($\mu$mol/L) | 0.002 | 0.001 | 2.315 | 1.002 (1.000, 1.004) | 0.128 |
| Serum RBP (mg/L) | 0.010 | 0.005 | 4.239 | 1.010 (1.000, 1.020) | 0.040$\dagger$ |
| Serum cystatin C (mg/L) | 0.602 | 0.144 | 17.392 | 1.825 (1.376, 2.421) | $<$0.001$\dagger$ |
| Serum uric acid ($\mu$mol/L) | 0.001 | 0.001 | 1.343 | 1.001 (0.999, 1.003) | 0.247 |
| eGFR (mL/min/1.73m^2^) | -0.020 | 0.004 | 25.721 | 0.980 (0.972, 0.988) | $<$0.001$\dagger$ |
| Triglycerides (mmol/L) | -0.046 | 0.056 | 0.673 | 0.955 (0.856, 1.066) | 0.412 |
| Total cholesterol (mmol/L) | -0.111 | 0.053 | 4.330 | 0.895 (0.806, 0.994) | 0.037$\dagger$ |
| LDL-C (mmol/L) | -0.177 | 0.080 | 4.884 | 0.837 (0.716, 0.980) | 0.027$\dagger$ |
| HDL-C (mmol/L) | -0.106 | 0.266 | 0.158 | 0.900 (0.534, 1.515) | 0.691 |
| Microscopic hematuria (Yes/No) | -0.290 | 0.236 | 1.504 | 0.748 (0.471, 1.189) | 0.220 |
| UPE (g/24h) | 0.005 | 0.024 | 0.050 | 1.005 (0.960, 1.053) | 0.823 |
| UAE (mg/24h) | 0.000 | 0.000 | 0.002 | 1.000 (1.000, 1.000) | 0.966 |
| Urinary IgG (mg/24h) | 0.001 | 0.000 | 7.962 | 1.001 (1.000, 1.001) | 0.005$\dagger$ |
| Urinary $\beta$2-MG (mg/24h) | 0.010 | 0.005 | 4.644 | 1.010 (1.001, 1.020) | 0.031$\dagger$ |

Abbreviations: B, coefficient; $\beta$2-MG, $\beta$2-microglobulin; BMI, body mass index; BUN, blood urea nitrogen; CI, confidence interval; DBP, diastolic blood pressure; DKD, diabetic kidney disease; eGFR, estimated glomerular filtration rate; ESR, erythrocyte sedimentation rate; FPG, fasting plasma glucose; Hb, hemoglobin; HbA1c, glycated hemoglobin; HDL-C, high density lipoprotein cholesterol; Hs-CRP, high-sensitivity C-reactive protein; IgG, immunoglobulin G; LDL-C, low density lipoprotein cholesterol; NDKD, non-diabetic kidney disease; OR, odds ratio; RBP, retinol-binding protein; SBP, systolic blood pressure; S.E., standard error of the coefficient; T2DM, type 2 diabetes mellitus; UAE, urinary albumin excretion; UPE, urinary protein excretion; Wald $\chi$^2^, Wald chi-square statistic. $\dagger$: *P*$<$0.05 versus NDKD.
